# Trans-ethnic genome-wide association studies: advantages and challenges of mapping in diverse populations

**DOI:** 10.1186/s13073-014-0091-5

**Published:** 2014-10-31

**Authors:** Yun R Li, Brendan J Keating

**Affiliations:** The Center for Applied Genomics, 1,016 Abramson Building, The Children’s Hospital of Philadelphia, Philadelphia, 19104 PA USA; Medical Scientist Training Program, Perelman School of Medicine, University of Pennsylvania, Philadelphia, 19104 PA USA; Department of Pediatrics, Perelman School of Medicine, University of Pennsylvania, Philadelphia, 19104 PA USA; Department of Surgery, Division of Transplantation, Perelman School of Medicine, University of Pennsylvania, Philadelphia, 19104 PA USA

## Abstract

Genome-wide association studies (GWASs) are the method most often used by geneticists to interrogate the human genome, and they provide a cost-effective way to identify the genetic variants underpinning complex traits and diseases. Most initial GWASs have focused on genetically homogeneous cohorts from European populations given the limited availability of ethnic minority samples and so as to limit population stratification effects. Transethnic studies have been invaluable in explaining the heritability of common quantitative traits, such as height, and in examining the genetic architecture of complex diseases, such as type 2 diabetes. They provide an opportunity for large-scale signal replication in independent populations and for cross-population meta-analyses to boost statistical power. In addition, transethnic GWASs enable prioritization of candidate genes, fine-mapping of functional variants, and potentially identification of SNPs associated with disease risk in admixed populations, by taking advantage of natural differences in genomic linkage disequilibrium across ethnically diverse populations. Recent efforts to assess the biological function of variants identified by GWAS have highlighted the need for large-scale replication, meta-analyses and fine-mapping across worldwide populations of ethnically diverse genetic ancestries. Here, we review recent advances and new approaches that are important to consider when performing, designing or interpreting transethnic GWASs, and we highlight existing challenges, such as the limited ability to handle heterogeneity in linkage disequilibrium across populations and limitations in dissecting complex architectures, such as those found in recently admixed populations.

**Electronic supplementary material:**

The online version of this article (doi:10.1186/s13073-014-0091-5) contains supplementary material, which is available to authorized users.

## Introduction

Large-scale genome-wide association studies (GWASs) have led to the discovery of thousands of genetic signals across the human genome associated with human diseases and quantitative traits [1]. These findings have led to significant advances, not only in identifying functional variants and in understanding how such genetic variants can affect disease risk, but also in our understanding of how selective pressures and natural selection have affected the human genome [2]. Although most GWASs originally focused on populations of European ancestry, `transethnic' studies that incorporate genotype data from more than one population or focus on replicating known associations in other ethnicities have become increasingly popular and have an important role in genomic medicine today. Using these transethnic analyses, several fine-mapping analyses have highlighted the newly recognized but essential role for regulatory and non-coding variants in disease biology and gene regulation. Understanding how coding and non-coding variants together can affect disease risk through such fine-mapping and resequencing efforts is arguably the most challenging and exciting area for genomic medicine today, because it offers opportunities for drug discovery or repositioning (by targeting specific mutations, signaling receptors or biological pathways).

Despite significant advances in high-throughput genotyping platforms, more powerful human genome reference panels and accurate imputation methods, major challenges remain. One is the apparent gap between the estimated disease heritability attributable to genetic factors (based on family and population genetics studies) and the small proportions of the total genetic heritability evident for most traits and common diseases explained through GWASs [3]. This gap, referred to as `missing heritability', remains a significant impediment, not only to understanding the role of genetic risk factors in human disease, but also for the disease-predictive utility of such genetic information - a much-espoused goal of genomics in the personalized medicine era. As such, the seeming incremental gain in disease or phenotype prediction based on this analysis of common human variation has been heavily criticized by many in the clinical community, as it remains unclear whether these results have significant clinical utility.

Various approaches have been proposed to test the models put forth by the genetics community to explain the observed missing heritability [4]-[6]. Rare variants, gene-environmental interactions [7], and other factors that can contribute to phenotypic heterogeneity probably contribute to disease heritability, as recently shown in the context of cancer [8],[9] and neuropsychiatric diseases such as autism and attention deficit hyperactivity disorder [10]-[12]. Because the frequencies of *bona fide* disease-causing genetic variants are known to vary between populations and because environmental exposures can also be altered, there has been much interest recently in the design and implementation of transethnic studies.

Furthermore, with the sheer numbers of individuals required to detect small to modest effect sizes, the bolstering of all populations available across large disease-analysis consortia is becoming more common, particularly in the study of quantitative traits where common international laboratory standards are used [6],[13]-[15]. Moreover, when designed properly, transethnic population findings enable a finer dissection of genetic architecture within a population. Specifically, it can be difficult to perform locus fine-mapping in intra-ethnic studies, as pinpointing the causal variant in the presence of strong linkage disequilibrium (LD) across a locus tends to be difficult, as compared with studying populations with limited LD in the same locus. This problem has been frequently observed at several loci originally identified from studies of European populations that have since been fine-mapped in Asian or African populations (Table 1).

**Table 1:** Landmark and innovative transethnic genetic association analyses*

Trait	Gene or locus	Platform	Comments	References
Type 2 diabetes	*TCF7L2*	Haplotype analysis	Replication of primary signal in WA population and fine-mapping of second independent signal showing positive selection in WA, EA and EUR cohorts; recently also replicated in large-scale meta-analysis over 39 studies	[42],[105]
Lipids (HDLC and TGs)	*ABCA1*, *LCAT*, *LPL*, *PON1*, *SERPINE1*	Candidate gene resequencing	Fine-mapping of known *LPL* gene association in AA with extreme lipid phenotypes, replication in WA, and showed stronger effect size of causal variants (local ancestry effects) as compared to EUR	[106]
End-stage kidney disease	*APOL1*	GWAS	Common variants in *APOL1* associated with resistance to *Trypanosoma* also confer risk for renal disease	[15]
Uric acid levels (serum)	*SLC2A9*	GWAS	Replication of a 263 kb association locus (identified in EUR) in an AA cohort enabled fine-mapping to a 27 kb shared region	[107]
Bilirubin levels	*UGT1A1*	GWAS	Replication of previously identified association in this locus in EUR and ASN cohorts using AFR population; also enabled fine-mapping to a functional, putatively causative variant	[108]
ALL	*CEBPE*, *PIP4K2A*, *ARID5B*	GWAS	Known risk-associated variants are more common in NA, confer greater risk and explain the higher observed risk of ALL in Hispanic children. Illustrates how disease risk analysis can shed light on disease associations in admixed populations with complex genomic architectures	[109]
T2D	*HNF1A*	Exome seq	High-throughput sequencing identified rare, novel missense mutation in a known locus associated with maturity-onset diabetes (MODY3); association is specific to Latino populations. Recently highlighted in a review on admixed population analysis	[82],[110]
Prostate cancer	15 EUR-specific, 7 multi-ethnic	GWAS	Large study encompassing over 40,000 cases and 40,000 controls in EUR, AFR, JPT, and Latino populations; multi-ethnic analyses help identify 7 new signals not found in EUR	[111]
BMI	*BRE*, *DHX34*, other	Custom genotyping platform	Metabochip analysis across about 30,000 AA individuals confirms 8 EUR BMI loci in AA, identified independent signal in known locus and identified two novel loci	[112]
Global gene expression levels	Multiple	Expression array	EUR, JPT and CHN populations show large variations in gene expressions due to differences in allele frequencies of common regulatory eSNPs, possibly explaining differences in complex disease risk	[113]
T2D	Multiple	GWAS meta-analysis	Landmark transethnic FE meta-analysis across nearly 27,000 cases from 5 ethnic minority populations identified 7 novel signals, enabled fine-mapping of 10 loci, and demonstrated evidence of heterogeneity compared with EUR studies using MANTRA software	[33]

*GWAS and other forms of genetic association studies have historically and recently provided important insights into disease-related loci. This table highlights a few notable examples, providing the study phenotypes, key associations (where specific), and details of the study including any unique approach used and the main findings/advances. *Abbreviations:* AA, African American; AFR, African; ALL, acute lymphoblastic leukemia; ASN, Asian; BMI, body mass index; CEU, Caucasoid; CHN, Chinese; EA, East Asian; eSNP, expression single nucleotide polymorphism; EUR, European; FE, fixed effects; GWAS, genome-wide association study; HDLC, high density lipoprotein cholesterol; JPT, Japanese; LD, linkage disequilibrium; NA, Native American; RE, random effects; T2D, type 2 diabetes; TG, triglycerides; WA, West African.

In this review, we highlight some of the key advances from the recent literature in which transethnic GWASs have been used for locus discovery, replication, fine-mapping or admixture mapping of causal variants associated with complex diseases. We also discuss advances and challenges in the use of transethnic GWASs by highlighting recently published software that apply new algorithms to boost the power of transethnic meta-analysis by leveraging LD information and the underlying differences in genetic architecture across disparate ancestral human genomes. In addition, we provide examples of recent studies that implement these methods and highlight their advantages and disadvantages over traditional GWAS meta-analytic approaches. Although our review is limited to disease-association traits, transethnic studies have also been used in other applications, such as the analysis of pharmacogenomics response [16]-[18] and of other phenotypic traits [19].

We conclude by noting the many challenges that remain in using samples from multiple diverse populations. Aside from limitations in sample sizes, with limited availability of genotyping and sequencing data from ethnic minorities, the ability to identify *a priori* appropriate study populations is difficult. For example, the currently available methods for performing transethnic meta-analysis still face limitations in power and also have limited ability to estimate joint effect sizes in the presence of effect heterogeneity.

## The need for transethnic genome-wide association studies

Transethnic studies are increasingly being used to increase study power by increasing the total study sample size. This is in part because there are limited sample sizes available for many diseases and because several consortia across the world have been established in countries whose populations are of diverse ancestries. The largest transethnic studies so far include studies of factors involved in metabolic and cardiovascular diseases, including high-density lipoprotein and low-density lipoprotein (LDL) levels [20], ischemic stroke and coronary artery disease [21] and blood pressure [22]; immune traits such as rheumatoid arthritis (RA) [23] and asthma [24]; neurocognitive and psychiatric diseases; and common oncologic diseases, including breast cancer [25] and prostate cancer [26].

Although a common goal in each of these large-scale transethnic GWASs is still disease/trait locus discovery, these studies also simultaneously make use of other features of transethnic study designs in four ways. First, they provide an independent replication sample set that can overcome concerns about sub-population or cryptic population stratification effects in single-population GWASs [27] and that can prioritize loci for secondary replication and sequencing studies [28]. Second, they boost study power by increasing the sample size. Third, they also strengthen the ability to evaluate the `common disease, common variant' hypothesis by demonstrating a common direction of effect for risk-associated alleles across populations when power or effect size is limited [29]. Fourth, they enable the identification of rare or causal variants by fine-mapping the association signals that are persistent despite major differences in LD structure across genetically diverse populations. Along the same lines, they can help point to expression quantitative trait loci (eQTLs or eSNPs) to identify functionally or mechanistically important regions (transcription factor binding sites, microRNA target sites or regulatory untranslated regions) that affect transcription rate, post-transcriptional or post-translational regulation or protein activity. Finally, they illustrate how selective pressure affects allele frequencies and transmission, when a given ancestral allele contribute to disease risk. This can be particularly fruitful when such risk alleles are carried by individuals from admixed populations.

## Replication and prioritization of GWAS candidates

One of the most common motivations for pursuing transethnic GWASs is to evaluate whether *bona fide* associations identified for a disease or trait in one population also affect other populations of different genetic ancestries. In the era of genomic medicine, the identification of such SNPs that can predict disease risk or therapeutic response is helpful in evaluating potential clinical or disease-predictive utility. Moreover, because GWAS association signals represent only a statistical correlation between genetic variations and disease or phenotype status, rather than causation, they are sensitive to sources of confounding and bias. Concerns about false positives are further amplified because of the large number of comparisons, as most standard GWAS platforms capture several hundred thousand to millions of variants and several tens of millions of variants following imputation.

Consequently, the initial goals of early transethnic studies had been to replicate the associations identified in one population in a second population with a distinct ancestry. At first these efforts aimed to directly replicate SNP-specific associations (by direct genotyping only the candidate SNP in a second population, rather than performing an independent GWAS), but it soon became apparent that achieving direct replication in an independent cohort posed significant challenges. Some SNPs have been consistently replicated across multiple ancestral populations - for example, the primary *TCF7L2* variant for type 2 diabetes (T2D) and the variant in the 9p.21 region for coronary artery disease. However, such consistent replications are likely to be the exception rather than the rule, because many disease or trait-associated SNPs reaching genome-wide significance do not directly replicate in studies of populations from a different ancestry. Although the *TCF7L2* and 9p.21 variants have moderate disease odds ratios (1.25 to 1.3), they have high minor allele frequencies (MAFs), which significantly aided their detection.

Although some initial putative associations are undoubtedly spurious (that is, attributable to population stratification or genotyping artifacts), the lack of direct replication could also be attributable to technical and biological factors, even for a true association [3],[30]. For example, there will be no transethnic replication if there is significant heterogeneity in the LD structure across different ethnic populations or if there is significant heterogeneity in the clinical phenotype or trait. In the former case, a major biological challenge comes when allele frequencies differ greatly across populations [31], as the ancestral allele frequency can also differ, for example, in HapMap European (CEU) versus African (YRI) populations. Consequently a given variant may be polymorphic or monomorphic in the second population, which makes directional and allele-specific replication challenging. Furthermore, a common variant that is less common or even rare in a replication population typically indicates that a greater sample size is needed to achieve comparable statistical power to detect a significant association [32],[33].

Nevertheless, many well-established SNPs have been replicated in transethnic studies. Notable examples include *PTPN22* in RA and inflammatory bowel disease [34]-[38], *INS* in type 1 diabetes [39],[40], *IL1RL1* in asthma [41] and *TCF7L2* in T2D [33],[42]. These results lend significant confidence and credibility to GWAS, because the replication of these lead index signals (essentially the most significantly associated signals, or the fine-mapped SNP with the strongest *P*-value in a candidate locus) in a population with significantly different LD structure overcomes the concern that a given signal is observed as a result of population stratification or other confounders (such as those introduced by environmental or geographical effects).

A recent large-scale review of published transethnic GWAS results across 28 diseases in European, East Asian and African ancestries [43] showed that a large proportion of the associations are caused by common causal variants that seem to map relatively close to the associated index genetic markers, indicating that many of the disease risk variants discovered by GWASs are shared across diverse populations. Even when power is insufficient to achieve statistically independent genome-wide significance, recent large-scale studies using summary-level data have shown unexpectedly high rates of directional consistency across transethnic GWAS signals [29].

As power is a function of both the strength of the association (effect size) and the MAF of the associated variant, limitations in transethnic replicability of variants resulting from limited allelic polymorphisms in a replicating population is a notable challenge. This is particularly the case in transethnic replication studies that incorporate resequencing data, which attempt to replicate findings of rare variants associated with disease. Recently, newer methods have been proposed for boosting the power of random effects models to provide multi-variant, gene-based testing that can be implemented in rare-variant transethnic association study designs [44].

Finally, despite these successes, new methods that can assess naturally occurring differences in population allele frequencies and LD structure are needed because it remains difficult to know which SNPs are expected or, conversely, not expected to be `replicable' given inherent genomic architectural differences. Such methods could help identify *a priori* a replication population of interest and also help reduce the frequency of performing `replication' studies in populations in which the associated variant is either non-polymorphic or too rare.

## Boosting power by large-scale transethnic meta-analyses

As the cost of genotyping has fallen precipitously since the first published GWAS (on age-related macular degeneration in 2005 [45]), independent efforts led by major genomics consortia, such as the Continental Origins of Genetic Epidemiology Network (COGENT), across multiple continents have since been published or are underway, investigating dozens of common heritable traits and diseases. A clear challenge of using transethnic GWASs to independently replicate new associations is the limited sample sizes, particularly if the variant was originally found in a genetically isolated population. Some studies have thus focused on finding out whether the directions of effects across replication cohorts are consistent, rather than attempting to replicate signals at genome-wide significance [29],[33],[46]. Although some consider a *P*_nominal_ <0.05 in a second cohort to be a replication signal, in most cases, when an independent GWAS has been performed it is more statistically rigorous to maintain a genome-wide significance threshold at *P* <5 × 10^-8^ in European populations [3],[30]. These efforts are further fueled by the challenge that the study power of any single cohort is limited given the high confidence threshold required to declare an association as genome-wide significance in the context of a large number of comparisons made in GWASs.

In the past few years, many global genomics consortia with enormous patient datasets have been used either in cross-continental mega-analyses directly or, more frequently, in summary statistic meta-analyses to better account for the wide ranges of genotyping platforms, genetic ancestry, environmental exposures, and other sources of sample heterogeneity. Two exemplary consortia that have published extensively using large transethnic cohorts include the T2D consortium and the RA consortium [23],[47]. Overall, however, attempts to use transethnic cohorts for direct replication of GWAS loci have met with only limited success [31],[48],[49].

## Methodological advances in transethnic meta-analysis

Although the publication of data from these transethnic studies is becoming increasingly frequent, these methods face several challenges, notably the presence of both genotype and phenotype heterogeneity. For example, not all SNPs found in one population are polymorphic in another, some disease-associated SNPs have vastly different MAFs across different populations [50],[51], and gene-environment interactions [52] and differences in study design or cohort recruitment could add to study heterogeneity. The need to appropriately adjust for population stratification in the presence of heterogeneity opposes the simultaneous need to optimize study power, a problem that remains highly challenging in the transethnic GWAS field.

Existing methods for cross-cohort meta-analysis assume, for the large part, one of two theoretical frameworks: fixed effects (FE) and random effects (RE) [53]-[55]. The former assumes that if a true association signal is identified in one cohort, that association will have a similar effect size in other cohorts. In contrast, RE models assume that effect sizes are highly variable, but that they follow a known (typically the normal) distribution. In the context of transethnic studies in which heterogeneity is to be expected, FE methods have limited utility, because of the typically high variance across studies: transethnic studies, in comparison with studies in a single ancestry, inevitably show higher inter-cohort heterogeneity.

Although in the presence of heterogeneity the RE model is more statistically sound, RE methods operate under a fairly conservative assumption that even null associations can have greatly varying effect sizes. Consequently, in these traditional methods, heterogeneity in the effects observed across populations results either in a down-estimate of the effect size because some populations do not show this association (when one obtains a mean estimate of effect), or in an overestimate of the standard errors that reduces the overall confidence of the association signal identified (by adjusting for heterogeneity). These are the main reasons that neither of these approaches are ideal when considering multiple, ethnically diverse cohorts together in a transethnic GWAS. Their advantages and limitations have been addressed thoroughly elsewhere [56],[57].

Two recent approaches, including alternate random effects (RE-HE) [56] and MANTRA [58], have been proposed to address some of the limitations met by traditional FE or RE models for meta-analysis. Both of these have been implemented in open-source software and are publically available. Central to both methods is the goal of optimizing study power when there is significant inter-study heterogeneity. Briefly, the approach taken by Han and Eskin [56] in developing the RE-HE model is based on the observation that RE methods have less power than traditional FE models because they assume an overly conservative model under the null [45]. Thus, by relaxing this overly conservative assumption, Han and Eskin demonstrated that the RE-HE model is more powerful than either traditional RE or FE methods when there is a true association but significant inter-study effect heterogeneity [56].

Although the RE-HE method is not specific to transethnic studies, it is clear that implementing this model would be particularly helpful. In contrast, Morris [58] introduced MANTRA specifically to address heterogeneity across studies in transethnic meta-analysis. The primary advance introduced in MANTRA is taking into account the expected differences in genetic architecture across different ethnicities in a transethnic study by using differences in the local LD structure across diverse populations [58]. MANTRA expects populations with similar genetic ancestries to have more closely matched effect sizes, while allowing for greater heterogeneity in the effects observed for more diverse populations. MANTRA has been shown to have greater power in both detecting shared associations and fine-mapping causal variants than FE methods, and where there is correlation between genetic similarity and similarities in effect sizes, MANTRA performs significantly better than RE.

These methods have been used successfully by a few transethnic and large-scale meta-analysis efforts, although their applications have been thus far limited to a few publications [7],[14],[33],[56],[59]. Future work using them along with functional data from population-specific studies (such as eQTLs and allele- and tissue-specific transcript expression) could help further advance these approaches in the era of large-scale integration of multiple `omics' resources. These methods have been compared directly against other meta-analysis methods in several recent reviews, including a thorough analysis by Wang *et al*. [57], who demonstrated that both RE-HE and MANTRA were superior to traditional approaches in transethnic meta-analysis, with RE methods having the poorest power. Specifically, the power and sensitivity of these methods in the context of known MAF and population genetic architectural heterogeneities have been taken into account.

Although MANTRA and RE-HE methods cannot be truly compared directly because the former uses a Bayesian framework, at the Bayes' factor significance threshold recommended by Morris [58], MANTRA seems to outperform RE-HE in nearly all instances except when there is no heterogeneity in effect sizes across studies [57]. MANTRA has been used in recent transethnic studies, including a landmark meta-analysis on T2D by the DIAGRAM consortium with over 76,000 individuals genotyped [33].

However, the use of these new approaches is still limited, and most recent studies have applied one or a combination of the traditional FE or RE meta-analysis models [60]-[63]. We recommend that studies consider implementing, alongside traditional methods, one or more of these newer, more powerful methods. In addition, it is crucial that for all such meta-analyses the author should assess and report a power calculation when discussing the presence or absence of independent transethnic replication. In many instances in which traditional methods are used, it is unclear whether the lack of significance in a replication cohort is the result of limited power or sample size in the presence of significant heterogeneity, or truly the absence of genetic association.

## Locus fine-mapping: identifying causal and functional variants in case-control and quantitative trait transethnic GWASs

An inherent advantage of transethnic studies is that demonstrating that signals are shared across multiple distant ancestral populations can help guard against false positives identified by GWASs due to population-stratification-related confounding. Although numerous methods have been identified in attempts to overcome such risks, they remain a challenge and concern, which is why independent replication, particularly in a second cohort, is still the gold standard in the GWAS community. Furthermore, because association signals in homogeneous populations are identified across a conserved LD block, it is not clear which SNP is the most strongly associated with a given phenotype, and consequently is most likely the functional or causal variant.

**Figure 1:**
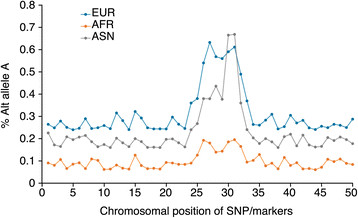
**Fine-mapping of candidate causal or functional SNPs by transethnic GWAS.** The graph shows the results of association testing (in the form of the allele frequencies) for a typical locus in three different populations. In the EUR population, many SNPs in the region are in close LD, leading to a significant signal for a wide set of SNPs. However, LD patterns in the ASN population are different, which enables finer mapping of the causal SNP as being the SNP with the strongest trait association. However, it is rarely obvious in advance which additional populations should be studied, as in some populations (such as AFR in this example) the locus might not be associated with the trait at all, because of epistatic interactions, phenotype heterogeneity, or low minor allele frequency/non-polymorphic markers across the locus. Data shown are based on simulation and do not reflect the result of any published or unpublished studies. Abbreviations: ASN, Asian; AFR, African; EUR, European.

Furthermore, in the past few years, the genomics community has shifted its focus from locus discovery to identifying casual or functional variants, in response to heavy criticisms of the limited utility of GWAS results and in an effort to better establish whether there is significant utility of such genetic information. Although most GWAS signals are found in non-coding regions of the genome (either intronic or intergenic regions), it is thought that some common association signals are proxies that `synthetically tag' the rarer causal or functional mutations in LD [64]. Based on these principles, deep resequencing around candidate loci followed by association testing to identify the most significant disease/trait-associated SNP within the candidate locus is commonly referred to as locus fine-mapping. In this approach, the top signal identified across different populations in a locus where the signal has been identified in both populations can help pinpoint the causal or functional variant of interest (Figure 1). Such methods have been used to successfully identify biologically plausible candidate gene mutations [65] and improve the total variance explained by identified loci by up to 50% [66], as has been shown for LDL.

Although resequencing techniques are becoming widely available and more economically feasible, genotyping is still advantageous in the study of variants with MAFs greater than 1 to 5%.

This is particularly true with the now widely available, high-density population-based genome references, such as the 1000 Genomes project and the ongoing UK-10 K and Genome Netherlands projects [67],[68]. To boost the power to identify functional or causal variants, several strategies have been implemented: directly increasing sample size and transethnic approaches. This area will likely benefit from additional development. For example, one question that remains controversial is whether a population-specific or mixed-population reference sequence panel should be used for genome imputation, to ascertain untyped markers when attempting to fine-map admixed populations or populations without a precisely matching reference panel [69]-[72].

Towards this goal, transethnic GWAS designs use naturally occurring differences in the LD patterns surrounding the locus of interest to help identify the likely causal or functional variants(s). Specifically, it is expected that the causal or functional variation would be associated with disease or trait status even in different populations in which the ancestral or derived haplotype frequencies differ significantly because of population drift or under selective pressures. Consequently, this allows the dissection of the key functional variant from other variants that are tagging signals on the same haplotype, because the non-causal tagging signals will be less likely to be preserved across diverse populations. This is particularly helpful, for instance, in using populations with more diverse haplotypes (such as African populations) to help refine signals from a less diverse group (such as European). Similarly, local ancestry analysis in admixture populations such as Mexican or Native American populations can also be helpful in refining a signal spanning a large LD block (see below).

Methods such as MANTRA, as discussed above, have also been effectively implemented in several transethnic fine-mapping studies - for example, across 14 central adiposity loci [59] and to discover and fine-map serum protein loci in European and Japanese cohorts [14]. Extension of MANTRA to additional cohorts and phenotypes will probably be fruitful because these newer algorithms have not yet been widely used to study transethnic cohorts. This is because most studies so far still use traditional meta-analysis frameworks to summarize transethnic association findings [41],[60],[62],[63],[73]. Several recent studies have shown that transethnic approaches to fine-mapping can improve the total variance explained across known association loci [15],[74]. A summary of the methods discussed above and example applications of these methods in landmark manuscripts are provided in Table 2.

**Table 2:** Methods, tools, literature reviews and resources*

Method or advance	Advances and limitations or main findings	References
MANTRA transethnic meta-analysis software	Replication of primary signal in WA population and fine-mapping of second independent signal showing positive selection in WA, EA and EUR cohorts. MANTRA is available as a suite of executables on request from the author [58]. Major limitation in that it cannot estimate a joint effect size even for the combined meta-analysis	MANTRA [58]; applications: adiposity loci [59]; quantification of serum protein [14]; T2D [33]
RE-HE random-effects method	RE and FE models in the context of a meta-analysis with significant heterogeneity have low power. By relaxing overly conservative parameters in RE analysis algorithms, RE-HE provides more power in the presence of inter-study effect heterogeneity. Metasoft is available as a package [114]; it provides a joint effect size estimate, but it is the same as the RE estimate	RE-HE algorithm [56]; applications: endometriosis [115]; bipolar disorder [18]; multi-tissue eQTLs [116]
Review on replicability of transethnic association signals	Comprehensive review of literature across 28 diseases in EA and EUR populations demonstrating high replicability, sharing of disease alleles and good correlation of effect sizes	[43]
Review on power gains in meta-analytical approaches	Simulation-based analysis demonstrating that a multi-ethnic study design provides non-trivial power gains, especially when AFR populations are used to examine low frequency alleles (MAF <5%)	[117]
Comparative analysis of FE, RE, RE-HE and MANTRA as a method for GWAS meta-analysis	Results show that both RE-HE and MANTRA are computationally efficient and robust methods in accounting for effect size heterogeneity while providing a boost in power when compared with traditional meta-analysis methods. Results are provided for both simulations and application to T2D datasets	[57]
Modified RE-HE for joint analysis of resequencing data for rare variant gene-based analysis	Extension of RE-HE to provide a more powerful (than traditional RE) method to perform rare-variant burden testing in a heterogeneous resequencing study sample	[44]

*Summary of innovative methods, applications and literature reviews as highlighted in the main text. We summarize the methodological advances, including those for meta-analysis, any significant or notable limitations, and for reviews. *Abbreviations:* AFR, African; ALL, acute lymphoblastic leukemia; EA, East Asian; eQTL, expression quantitative trait locus; EUR, European; FE, fixed effects; GWAS, genome-wide association study; LD, linkage disequilibrium; MAF, minor allele frequency; RE, random effects; RE-HE, alternate random effects; T2D, type 2 diabetes; WA, West African.

## Using admixture mapping in transethnic study designs

One of the major observations from transethnic studies is the limited direct replicability of signals identified in one population associated with a given phenotype in a second population of differing ancestry. However, as demonstrated elegantly by Wijmenga and colleagues [75] for four well-studied GWAS traits, although specific variants might not be shared between populations, when one also considers markers in close proximity to the originally identified markers, the replicability of variants across populations is relatively high.

Thus, although genetic studies of a range of phenotypes across different populations have not yielded associated loci common to all or even the majority of investigated ancestry groups, this could be for a variety of reasons independent of whether this is a truly shared risk- or phenotype-associated variant: population-specific variants, differences in allele frequencies, different patterns of LD across respective populations, and/or low statistical power from modest sample sizes, as discussed above.

One traditional technique used to identify disease-association or phenotype-associated regions of the genome, which was used and advanced before the advent of high-density genotyping platforms and the GWAS era, was the use of ancestry-informative markers in admixture mapping [76],[77]. Admixture mapping using populations that have recently undergone gene flow from two ancestrally isolated populations, such as African Americans, is a very powerful method to detect disease variants where there are substantial allele frequency differences in the ancestral populations [32],[41],[57],[58]. In broad terms, the goal of an admixture study 000is to identify the risk-associated allele (for a given disease) based on the likelihood of observing an association between a given ancestral allele(s) with disease risk [78],[79]. Both case-control and case-only study designs are feasible, with the latter adding flexibility and reducing the need for a large control sample size, which can be particularly difficult to ascertain in admixed populations.

**Figure 2:**
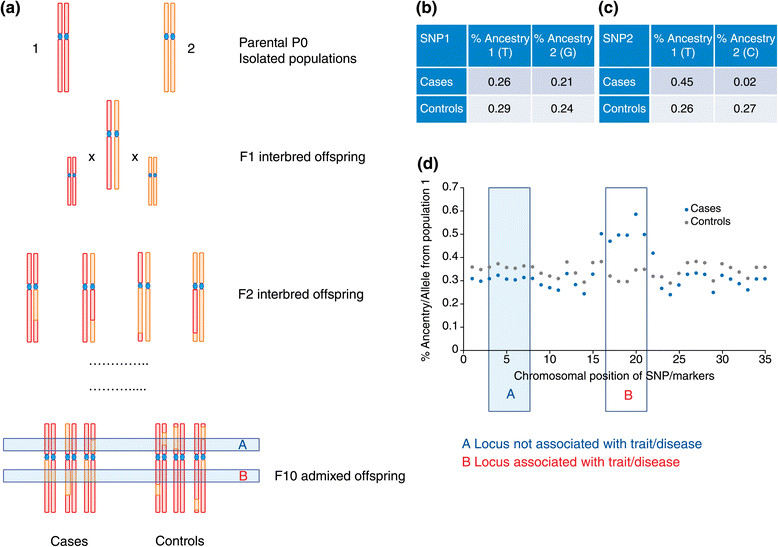
**Theoretical basis of admixture GWAS study designs. (a)** Populations 1 and 2 are two parental populations in which there has been no gene flow historically. When these populations interbreed the subsequent F1 population includes heterozygotes. Over the course of 5 or 10 generations the chromosome of any given F_n_ population offspring will include a combination of parental chromosomal `bands'. Some loci are associated with a disease (such as B) and others are not (such as A). **(b, c)** In a typical GWAS, association testing identifies whether a given allele (such as T at SNP2) is associated with increased risk for having a disease; this is shown as allele frequencies in the table. **(c)** If the ancestral frequency of T at SNP2 is different in two parental populations (1 and 2) and if it is associated with disease, then the population with higher frequencies of this allele will also have higher risk for disease. One can thus expect to observe higher incidences of disease in individuals carrying the T allele and also higher incidence of disease in individuals from population 1, in which the T allele is more frequent. This is the premise of admixture association studies. By ascertaining local ancestry one can determine if an allele that is much more common in one population may be associated with disease risk. In **(b)**, in a locus with no evidence of association with disease, admixture analysis would find that the minor allele frequencies (and percentages of individuals of either ancestral populations) do not differ between cases and controls. **(d)** Graph of the allele frequencies along the genome. The relative frequency of the allele from population 1 differs between the cases and the controls only at the locus associated with the disease/phenotype. Thus, in admixed populations, by determining the local ancestry in the cases versus controls, one can determine if there is an association between an allele associated with ancestry and disease liability.

The theoretical framework for admixture-based genetic mapping analysis is complex and beyond the scope of this review, but it is summarized briefly in Figure 2 (see also several reviews [78]-[84]). The most commonly used method is mapping by admixture linkage disequilibrium (MALD), which uses the fact that the prevalence of the disease studied is considerably different between ancestral populations of the admixed cohort [78],[79],[85].

In contrast to transethnic analyses, in which isolated populations are investigated, admixture GWASs can help avoid the bias introduced by confounding in GWASs in the presence of mild to moderate degrees of population stratification. Traditional approaches to handling population stratification, typically by adjusting for differences in global ancestry, are challenging and often insufficient in either ethnically diverse or mixed ancestry populations (for example, Hispanic or African American cohorts), given that efforts that focus on simply adjusting for global ancestry are often insufficient or under-powered [83],[86],[87]. Methods for local ancestry adjustments have been put forth as powerful alternatives to controlling for population substructure in association testing of admixed cohorts [81],[82], but this has recently been challenged by work from Shriner *et al*. [88], who proposed a potentially more powerful joint approach to admixture mapping and association testing that accounts for both global and local ancestry.

Alternatives to adjusting for ancestry differences by using linear mixed model approaches, which have gained popularity recently, have only been applied so far to closely related populations, not to transethnic GWASs. Consequently, directly merging genotypes from either ancestrally divergent populations or those that have undergone varying degrees of admixture using traditional association testing frameworks (such as global ancestry adjustment using principle component or multi-dimensional scaling) to adjust for population substructure does not sufficiently control for the risk of confounding [77],[81],[89],[90]. An inherent advantage of admixture mapping is that it bypasses this challenge because its goal is to firstly assign each allele (risk versus protective) to the ancestral population, and secondly test if there is a statistically significant overrepresentation of the allele from one ancestral lineage across cases versus controls [91].

Admixture mapping approaches, which uses significantly fewer tests across the genome, have been successfully used to study several traits and phenotypes, including blood pressure phenotypes in African Americans, for which no robust associations had previously been observed using conventional GWAS approaches [92]. Admixture mapping has also been used to identify loci contributing to various complex traits and diseases, including body mass index, multiple sclerosis, cholesterol levels and focal segmental glomerulosclerosis [93]-[97]. These studies have gained much clinical and epidemiological attention, in part because many of the investigated phenotypes and diseases occur at unexpected higher rates in admixed populations, such as Native Americans, African Americans and Latin Americans [85].

## Conclusions and remaining challenges

As the cost of genotyping and high-throughput sequencing technologies continues to drop, consortium-driven worldwide GWASs of complex diseases and phenotypes will probably continue to expand to ever larger cohorts, additional phenotypes and wider ethnic groups. In addition, coupled with current deep phenotyping and electronic medical record mining efforts, genomic medicine is entering an exciting era of phenomics and phenome-wide association studies (PheWASs), in which characterization of genetic and environmental effects across all traits and diseases might be within reach. Applying the methods discussed here for transethnic GWASs to PheWASs could be powerful, given the known stratification of related phenotypes and disease risk among ethnic groups.

Without a doubt, new findings from transethnic studies will enrich our understanding of several issues. First, the degree to which genetic associations are shared or population-specific in the presence of either shared or disparate genetic architecture; second, how architectural differences in LD patterns might affect the pattern of genetic association; and third, whether ethnically stratified disease prevalence is directly attributable to genetic or gene-environment interactions. New methods, such as MANTRA and RE-HE, as discussed here, offer more robust and better powered approaches to performing transethnic meta-analyses.

As the number of GWASs using transethnic and admixed populations increases, they present new opportunities for novel study designs using linkage information at either the variant level or the higher gene or pathway levels. However, numerous challenges remain for transethnic studies. Specific association markers typically demonstrate limited replicability in genetically distant cohorts and it is usually not known *a priori* which loci should have a good chance of being shared versus being population-specific. Nor is it clear which populations (including admixed ancestries) should be investigated to optimize the chance for locus discovery versus fine-mapping.

Wijmenga and colleagues, in their review of existing literature-reported transethnic GWAS replication rates across different study populations [75], observed that the replication rate of loci is high whereas that of individual SNPs is low. They concluded that many reports of non-replication in transethnic studies result from studies that are limited by differences in genetic architecture (some markers are non-polymorphic or rare in other populations) but not by the fact that these are not biologically conserved shared loci. To overcome this challenge, they advised the use of pathway- and gene-based methods [75]. Although not yet available, recently advanced gene- and pathway-based methods for GWAS are likely to be easily applied to transethnic datasets and to require little additional method development [98]-[100].

Another relevant question that has not been thoroughly explored is whether specific populations are more amenable or useful in a transethnic or admixture analysis; identifying optimal methods to answer this question in a locus-specific manner will be difficult. Some methods have been proposed: constructing marker panels for admixture studies using an information-theory-based measure, the expected mutual information score [85]; identifying markers that are most likely to be fine-mappable by transethnic study designs using LD information [101]; and identifying populations in which LD variations are optimal for transethnic [92] or admixture study designs [102]. Finally, Yang and Visscher and colleagues [103] recently described a linear mixed model to estimate the genetic variance explained by genome-wide markers as a method for estimating disease and trait heritability based on common SNPs. This has been extended by Coram *et al*. [20] to consider admixed populations. The proposed admixture-adjusted measures for trait and disease heritability will probably have broad applications.

Finally, work has also been done to examine how information on LD structure differences across ethnically diverse populations, and variant molecular function, can be used in a Bayesian framework to improve the power of association testing [104]. Although much work remains to be done to maximize the power of such transethnic and admixture population-based GWAS designs, it is clear that making use of this information will be important in both locus discovery and replication in non-European ancestral populations and in the identification of functional or mechanistic variations in the post-GWAS era.

## Authors’ original submitted files for images

Below are the links to the authors’ original submitted files for images. Authors’ original file for figure 1 Authors’ original file for figure 2

## Abbreviations

eQTL: Expression quantitative trait locus
eSNP: Expression single-nucleotide polymorphism
FE: Fixed effects
GWAS: Genome-wide association study
LD: Linkage disequilibrium
MAF: Minor allele frequency
RE: Random effects
RE-HE: Alternate random effects
SNP: Single-nucleotide polymorphism
T2D: Type 2 diabetes
