# Supplementary figures and images for: Trans-ethnic genome-wide association studies: advantages and challenges of mapping in diverse populations
Source: Genome Med. 2014 Oct 31;6:91. doi: 10.1186/s13073-014-0091-5 (PMC4254423; doi:10.1186/s13073-014-0091-5)

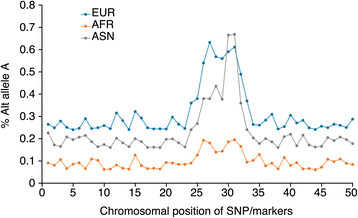

Supplement: Supplementary file 1 — Authors’ original file for figure 1 [file 13073_2014_91_MOESM1_ESM.gif]

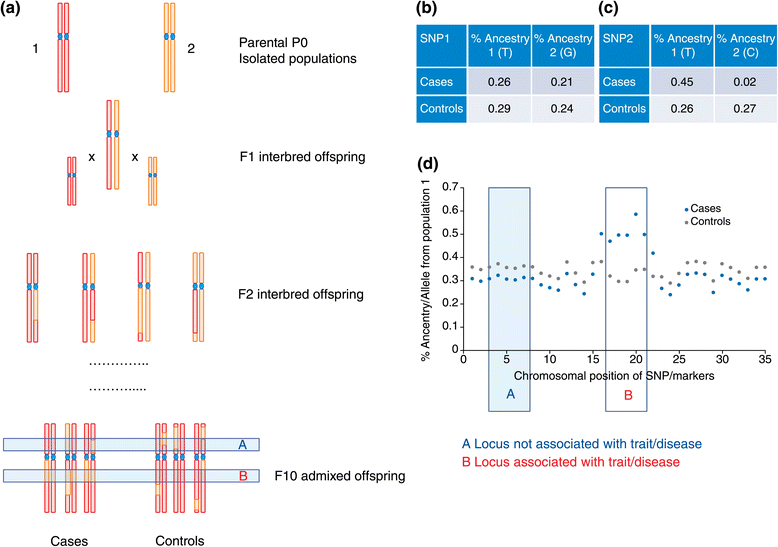

Supplement: Supplementary file 2 — Authors’ original file for figure 2 [file 13073_2014_91_MOESM2_ESM.gif]
